# An innovative interprofessional education simulation for athletic training and prelicensure nursing students: Development, implementation, and student perspectives

**DOI:** 10.1111/nuf.12825

**Published:** 2022-10-29

**Authors:** Jacqueline Vaughn, Robin Cunningham, Lindsey H. Schroeder, Colette Waddill, Matthew J. Peterson, Mia Rose Gambacorta, Stephanie Sims

**Affiliations:** ^1^ School of Nursing, College of Health & Human Sciences University of North Carolina Wilmington Wilmington North Carolina USA; ^2^ School of Health and Applied Human Sciences University of North Carolina Wilmington Wilmington North Carolina USA

**Keywords:** education, interprofessional education, simulation

## Abstract

**Background:**

The purpose of this article is to describe the development, implementation, and evaluation of a Simulation Interprofessional Education (Sim‐IPE) activity for healthcare students from different disciplines (athletic training [AT] and nursing). The objective for the Sim‐IPE activity was to engage AT and prelicensure nursing students in a realistic healthcare scenario to enhance knowledge about one another's profession, develop interprofessional skills, collaborate with one another, and communicate effectively as a team as they performed care.

**Methods:**

This mixed methods study employed a one‐time posttest design for a convenience sample of AT and prelicensure nursing students following a simulation intervention. Students completed the Student Perceptions of Interprofessional Clinical Education‐Revised (SPICE‐R) survey and answered open‐ended response questions.

**Results:**

Thirteen students (*N* = 13) from Cohort 1 and 12 students (*N* = 12) from Cohort 2 completed the SPICE‐R survey. Most students strongly agreed/agreed for each of the SPICE‐R survey questions. Qualitative findings indicated the students positively perceived the Sim‐IPE activity as it helped them discover the value of interprofessional patient care.

**Discussion:**

The quantitative findings indicated that the students found the Sim‐IPE an effective learning methodology to achieve the objectives while the qualitative findings gave further insight into the students' perceptions of interprofessional teamwork and the value of the prebrief session conducted before the simulation. The findings will inform future Sim‐IPE activities involving additional groups of healthcare students.

## 1 BACKGROUND

Interprofessional education (IPE) and collaboration have been established as essential components in healthcare education programs needed to prepare students for their future roles.^1^, ^2^ Engaging students in IPE can increase knowledge, foster competency, and strengthen skills for interacting with other healthcare workers.^3^, ^4^ Research shows effective interprofessional collaboration improves healthcare quality and patient safety and reduces healthcare costs.^5^, ^6^, ^7^

Simulation is a well‐studied, evidence‐based approach used to educate healthcare students.^8^, ^9^, ^10^ Simulation IPE (Sim‐IPE) is an experiential learning methodology that promotes student interaction, collaboration with other professions, team‐based decision‐making, and thus, enhances students learning from and with each other.^11^, ^12^ Sim‐IPE fosters the assimilation of new knowledge, skills, and attitudes which can enhance clinical competency used in future practice.^13^, ^14^

Many healthcare disciplines have developed standards for IPE. For example, in nursing education, the Healthcare Simulation Standards of Best Practice: Simulation Enhanced IPE is used to guide IPE activities.^15^ Additionally, the Commission on Accreditation of Athletic Training Education (CAATE) updated their standards for professional entry‐level athletic training (AT) programs in 2020.^16^ The new AT educational standards emphasize interprofessional collaboration while also reflecting the IPEC core competencies utilized in interprofessional simulation and standardized patient (SP) encounters.^16^

## 2 PROBLEM

While medical student and nursing student IPE is well documented in the literature, other healthcare disciplines are often less addressed.^17^ Our university has a college of health and human services which includes schools of Nursing, Social Work, and Health and Applied Human Sciences. This rich repository of healthcare students provides an opportunity to incorporate IPE into curricula and engage students across multiple academic programs.

This pilot Sim‐IPE activity targeted two healthcare disciplines, AT and prelicensure nursing students. The literature on the collaboration of these two student groups is sparse but growing.^17^ This article describes the development, implementation, and evaluation of a pilot Sim‐IPE for AT and nursing students. The findings will inform future Sim‐IPE activities involving additional groups of healthcare students. The objective for the Sim‐IPE activity was to provide a realistic healthcare scenario for students from different disciplines to learn about one another's profession, collaborate with one another, and communicate effectively as a team as they performed patient care.

### 2.1 Evidence‐based framework

The core competencies for interprofessional collaborative practices, created by the Interprofessional Education Collaborative (IPEC)^18^ served as the foundation for the Sim‐IPE. IPEC defines interprofessional collaboration through four core competencies: (a) values/ethics for interprofessional practice, (b) roles/responsibilities, (c) interprofessional communication, and (d) teams and teamwork.^18^, ^19^ The pilot Sim‐IPE incorporated the four core competencies in the simulation with the goal to prepare the healthcare students for interprofessional practice and collaboration.

## 3 METHODS

### 3.1 Study design

This mixed methods study employed a one‐time posttest design for a convenience sample of AT and prelicensure nursing students following a simulation intervention. This nonexperimental Sim‐IPE was reviewed and approved by the Institutional Review Board at the university.

### 3.2 Participants

Twenty graduate‐level AT students and 12 prelicensure nursing students from a university in the Southeastern United States participated. The AT students consisted of 11 first year‐students (Cohort 1) and 9 second‐year students (Cohort 2). Participation in the Sim‐IPE was mandatory for the AT students as it was part of their semester clinical course requirement. Prelicensure nursing students ranged from the second semester through the fifth semester and participated voluntarily. Nursing students were recruited via email and were assigned to either Cohort 1 or 2 depending on their availability.

### 3.3 Simulation design

The Sim‐IPE was created by faculty from the Athletic Training Program and School of Nursing. The simulation was designed using the Health Care Standards of Best Practice.^8^ Faculty from the two disciplines met biweekly over 3 months to outline the simulation and develop student objectives and outcomes. Two Sim‐IPEs were designed to accommodate the knowledge level of the AT learners (1st‐ vs. 2nd‐year students). The initial draft of the simulation was developed, then iteratively revised by the faculty.

**Figure 1:**
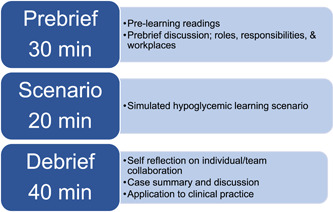
Study schema [Color figure can be viewed at wileyonlinelibrary.com]

The Sim‐IPE consisted of three faculty‐facilitated phases; a prebrief session, the simulation scenario, and a debrief session (Figure 1).

### 3.4 Prebrief session

Prebriefing, including preparation and briefing activities are considered standard of best practice and provide essential elements for achieving optimal student learning.^20^ The AT and nursing students had limited or no prior Sim‐IPE experience thus preparation materials were assigned before the activity. Prereadings included (1) two faculty‐selected healthcare IPE publications to familiarize students with IPE; (2) National Athletic Trainers' Association Position Statements; (3) an IPEC competencies summary sheet, and (4) a summary of the TeamSTEPPS Introduction, Situation, Background, Assessment, Recommendations (ISBAR) communication tool.^21^ These materials allowed the students to be prepared for the Sim‐IPE in effort to optimize successful learning outcomes.^20^

The briefing conducted immediately before the Sim‐IPE incorporated an orientation to the objectives, roles, and expectations for the students.^20^ Students were oriented to aspects of the experience to help them achieve the objectives: scenario, equipment, SP, and the scenario environments.

The briefing provided time to introduce the Sim‐IPE in the context of the IPEC competencies. Students collaborated during the briefing session by introducing themselves and sharing information about their respective disciplines. Students discussed their professional identity, roles, responsibilities, and major areas of work. This time allowed them to learn about one another's professions and plan for collaborative team‐based interactions. Students were encouraged to engage with classmates and ask questions about how one another's profession influenced care.

### 3.5 Simulation scenario (Cohort 1 and Cohort 2)

Ninety‐minute Sim‐IPE events were scheduled and implemented over two afternoons (one for Cohort 1 and the other for Cohort 2). Each 90‐min session was conducted three times to allow all students to participate and consisted of a prebrief, simulation scenario, and debrief. Each session consisted of two to three AT students and one to three nursing students. The 20‐min simulation scenario consisted of an SP actor playing the role of a student‐athlete “patient” who collapses in response to a hypoglycemic event. The scenario began at a simulated college cross‐country event. The SP was warming up for the race using a stationary bicycle when the signs and symptoms of hypoglycemia emerged. For Cohort 1, the SP portrayed mild signs of hypoglycemia, confusion, irritability, and dizziness before collapsing. For Cohort 2, who were further advanced in the program, the SP experienced severe signs of hypoglycemia including a seizure. The AT students performed the primary assessment on the patient, stabilized the patient, then transported the patient to the simulated medical tent staffed by the nursing students. The AT students gave a handoff report to the nursing students using the ISBAR communication tool. The nursing students conducted a primary assessment and stabilized the athlete while awaiting paramedic transport to a hospital emergency department. The scenario ended with the nursing student giving a condition update using the ISBAR tool to communicate assessment findings to the healthcare provider played by a faculty member.

### 3.6 Debrief session

AT and nursing faculty cofacilitated the debrief using the “Promoting Excellence And Reflective Learning in Simulation” (PEARLS) framework for debriefing.^22^ PEARLS is an evidence‐based effective framework that incorporates three educational strategies, (1) learner self‐assessment, (2) facilitating focused discussion, and (3) providing directive feedback and/or teaching.^22^, ^23^ Students were guided through the process of summarizing the scenario events, reflecting on their performance, highlighting the interprofessional skills used, and assimilating knowledge gained from participating in the Sim‐IPE.

### 3.7 Data collection instrument

Students completed the Student Perceptions of Interprofessional Clinical Education‐Revised (SPICE‐R) survey and open‐ended reflective questions at the end of the Sim‐IPE. The SPICE‐R survey was used to assess the AT and nursing students' attitudes toward interprofessional healthcare teams, their roles, collaboration, and the team approach to care. This is a validated and reliable (Cronbach's *α* = .86) instrument designed to evaluate IPE curricula among healthcare students.^1^, ^4^, ^24^ The survey consists of 10 questions and responses are captured using a five‐point Likert scale (1 = *strongly disagree*, 5 = *strongly agree*).^1^ The SPICE‐R Instrument evaluates three factors of interprofessional education: interprofessional teamwork and team‐based practice (items 1, 5, 6, and 8–10), roles/responsibilities for collaborative practice (items 2 and 7), and patient health outcomes from collaborative practice (items 3 and 4).^1^, ^24^ The SPICE‐R score is the sum of the 10 responses with a minimum possible score of 10 and a maximum possible score of 50.^1^ Additional open‐ended reflective questions developed by the faculty were used to obtain more detailed feedback on students' perspectives of the simulation and their prior experience with IPE. Reflective practice is an evidence‐based approach that healthcare professionals employ to critically evaluate and learn from experiences.^25^, ^26^ The academic and clinical performance of the students during the simulation was not formally evaluated as this was a formative Sim‐IPE experience.

### 3.8 Data analysis

Univariate, descriptive procedures included counts (*N*) and proportions (%). SPICE‐R response data were summarized by student cohort. All quantitative data were analyzed and visualized using SAS JMP Pro version 15 (SAS Institute).

Thematic analysis was used to examine responses to the open‐ended questions.^27^ The analysis team consisted of four coauthors who analyzed the qualitative data beginning with familiarizing themselves with the data and creating an audit trail. Initial codes were independently generated then reviewed and refined in team meetings. An initial coding guide was created by condensing similar codes. The analysis team then recoded the data using the guide and compared their second coding for consistency. After the final coding of the data, the analysis team identified a primary theme and subthemes characterizing the data into salient points.

## 4 RESULTS

Thirteen students (*N* = 13) from Cohort 1 and 12 students (*N* = 12) from Cohort 2 completed the SPICE‐R survey. Both cohorts were comprised mostly of women (~60%) and AT students (77% Cohort 1; 67% Cohort 2) (Table 1). The other student discipline was comprised of nursing students. Most students strongly agreed/agreed for each of the SPICE‐R survey questions (Table 2). One student responded “strongly disagree” to each survey question, however, there were no open‐ended responses that gave additional insight into these responses.

**Table 1:** Student demographics

	Cohort 1 (*N* = 13)	Cohort 2 (*N* = 12)
Gender, *N* (%)		
Women	8 (62%)	7 (58%)
Men	5 (38%)	5 (42%)
Discipline, *N* (%)		
Athletic Training	10 (77%)	8 (67%)
Nursing	3 (23%)	4 (33%)

**Table 2:** Student perceptions of interprofessional clinical education‐revised

	Strongly agree		Agree		Neutral		Disagree		Strongly disagree	
	Cohort 1 *N* (%)	Cohort 2 *N* (%)	Cohort 1 *N* (%)	Cohort 2 *N* (%)	Cohort 1 *N* (%)	Cohort 2 *N* (%)	Cohort 1 *N* (%)	Cohort 2 *N* (%)	Cohort 1 *N* (%)	Cohort 2 *N* (%)
Working with students from another health profession enhances my education.	12 (92.0)	10 (83.0)	1 (7.7)	1 (8.3)	0	0	0	0	0	1 (8.3)
My role within an interprofessional healthcare team is clearly defined.	8 (62.0)	7 (58.0)	3 (23.0)	3 (25.0)	0	1 (8.3)	1 (7.7)	0	1 (7.7)	1 (8.3)
Health outcomes are improved when patients are treated by a team that consists of individuals from two or more health professions.	12 (92.0)	8 (75.0)	1 (7.7)	2 (17.0)	0	0	0	0	0	1 (8.3)
Patient satisfaction is improved when patients are treated by a team that consists of individuals from two or more health professions.	10 (77.0)	8 (75.0)	3 (23.0)	2 (17.0)	0	0	0	0	0	1 (8.3)
Participating in educational experiences with students from another health profession enhances my future ability to work on an interprofessional team.	13 (100)	11 (92)	0	0	0	0	0	0	0	1 (8.3)
All health professional students should be educated to establish collaborative relationships with members of other health professions.	12 (92.0)	11 (92.0)	1 (7.7)	0	0	0	0	0	0	1 (8.3)
I understand the roles of other health professionals within an interprofessional team.	6 (46.0)	7 (58.0)	5 (38.0)	3 (25.0)	1 (7.7)	1 (8.3)	1 (7.7)	0	0	1 (8.3)
Clinical rotations are the ideal place within their respective curricula for health professional students to interact.	8 (61.5)	5 (42)	2 (15.0)	6 (50.0)	2 (15.0)	0	1 (7.7)	0	0	1 (8.3)
Health professionals should collaborate on interprofessional teams.	11 (84.6)	9 (75)	2 (15.6)	2 (16.7)	0	0	0	0	0	1 (8.3)
During their education, health professional students should be involved in teamwork with students from other health professions to understand their respective roles.	10 (76.9)	10 (83.3)	3 (23.1)	1 (8.3)	0	0	0	0	0	1 (8.3)

### 4.1 Thematic findings

**Figure 2:**
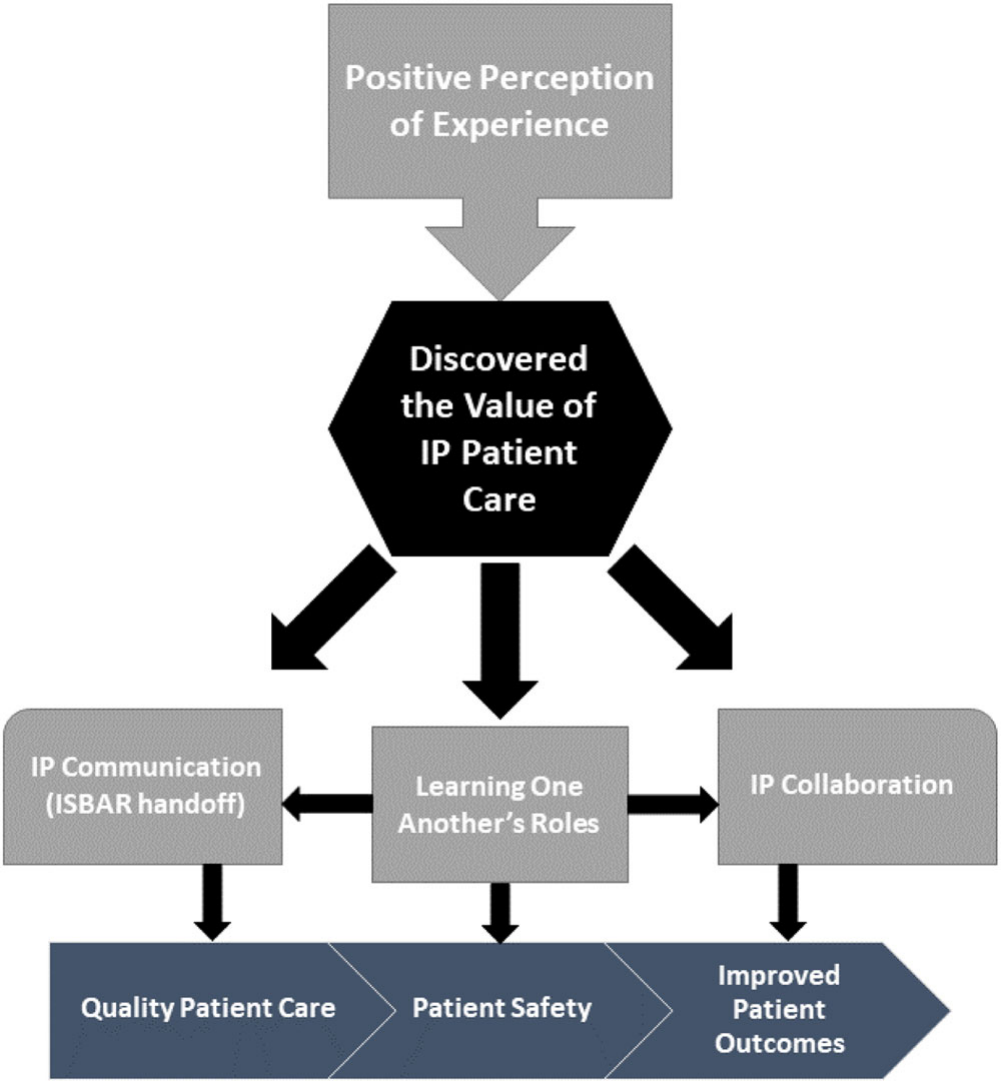
Thematic map [Color figure can be viewed at wileyonlinelibrary.com]

Following the SPICE‐R survey, students were asked to respond to open‐ended questions describing what they learned from the Sim‐IPE experience and its importance to their future healthcare careers. Qualitative analysis was used to provide deeper insight into the students' experience and their thoughts about interprofessional patient care. From these responses, one overarching theme was identified; students perceived the interprofessional experience positively as it helped them discover the value of interprofessional patient care. Subthemes that further illuminated the value of interprofessional patient care included the importance of interprofessional communication and collaboration and learning about one another's healthcare roles. Additionally, students recognized the impact that these subthemes had on the quality of patient care, patient safety, and patient outcomes (Figure 2).

### 4.2 Themes and subthemes

#### 4.2.1 Positive perception

Many students described positive perceptions related to their experience with the Sim‐IPE activity. One student commented “It's a great experience to become involved with other disciplines and to build a relationship with them,” while another responded, “It was a great experience.” Other students perceived the experience as positive since it impacted their knowledge, “I really enjoy working on an interdisciplinary team, it enhances my clinical knowledge,” and another noted, “It's an excellent learning experience.”

#### 4.2.2 Importance of interprofessional communication

The students also described how the experience helped them think about the importance of interprofessional communication for patient safety, specifically during a patient handoff with one noting “Being able to understand the information nursing needs from AT for the patient to get the best care was beneficial.” Another student commented on the structured communication tool, ISBAR, “I thought seeing an interdisciplinary team work together showed how important ISBAR is when conveying information.”

#### 4.2.3 Importance of interprofessional collaboration

Students commented on how participation in the Sim‐IPE helped develop a better understanding for how collaboration with other professions can enhance patient care and lead to improved patient outcomes. One student reflected “Working on an interdisciplinary team allows for the best collaborative plan to come about through the ideas of all disciplines.” Another student remarked, “I learned more about what nurses do in the real world and how we can work together to best help the patients.”

#### 4.2.4 Learning about one another's roles

In their responses, students expressed their thoughts on how participating in this experience helped them to gain a deeper understanding and new appreciation of other healthcare fields' roles and responsibilities. For example, students noted “I learned more about what nurses/athletic trainers do,” and “what nurses are responsible for.”

Another student commented, “I was with nursing students, and I got a view into their field and also how to interact with nurses.”

In addition to learning about one another's roles one student commented that the sim‐IPE gave them the opportunity to teach others about their profession's roles, “‘*the Simulation’* let us show them what we do as athletic trainers to advocate for our profession.”

## 5 DISCUSSION

Planned interprofessional education should strive to allow students from various healthcare programs to learn about, from, and with each other. Several studies have shown that students who participate in IPE activities report increased confidence, improved attitudes, and enhanced communication skills.^1^, ^2^, ^4^ Simulation‐based IPE offers multidisciplinary students the opportunity to actively engage with one another in meaningful, practical ways to facilitate learning and prepare them for practice.^1^, ^4^, ^17^, ^28^ It also affords educators the opportunity to incorporate varied/client patient populations and health concerns that students may not experience during their clinical education. When introducing these educational experiences into a program, educators can draw from their own experiences when they engaged with other healthcare professionals during clinical practice. By participating in the Sim‐IPE, students demonstrated interprofessional collaboration and communication in the medical tent where AT students gave a patient handoff using the ISBAR communication tool while students from both groups worked together to transfer the patient to the stretcher.

The SPICE‐R tool was used to assess the students' perceptions toward interprofessional healthcare teams, roles, collaboration, and teamwork after the interprofessional simulation. Most of the students strongly agreed or agreed that working with other interprofessional students enhanced their education and will affect their future ability to work on interprofessional healthcare teams. The qualitative findings gave deeper insight into these findings and highlighted their positive perceptions. Students detailed what they learned about one another's professions and roles and how this benefits their learning. An additional positive perception perceived by the students was that the Sim‐IPE experience broadened their perspective by exploring different viewpoints through discussion with other students. Our results suggest embedding Sim‐IPE experiences into healthcare students' curriculum fostered positive perceptions of IPE by allowing them to experience firsthand the benefits of teamwork, collaboration, and effective communication. This positive perception of IPE may influence future healthcare interactions.

The IPEC competencies emphasize collaboration and communication as essential elements of interprofessional work.^18^ Participating in the Sim‐IPE gave students the opportunity to actively engage with one another and practice skills to develop these competencies and thus recognize their importance. Importantly, the qualitative findings from this study indicate that the students were able to link these essential elements to quality patient care and improved patient outcomes. Their comments related to communication highlight the importance of what needs to be communicated to create an effective handoff to enhance patient safety (i.e., using ISBAR or other structure communication method). Their comments about collaboration illustrate the importance of teamwork and its impact on patient care and team functioning.

The Sim‐IPE prebrief was intentionally used to facilitate students' discussion and increase their knowledge of one another's roles and practice thus promoting IPEC competencies 1 and 2, Values and Ethics and Professional Roles and Responsibilities. The qualitative findings suggest the Sim‐IPE created conditions that promoted the development of positive attitudes for interprofessional collaboration and broadened their perspectives of other professions. Students noted they found the prebrief's initial introduction and discussion of one another's roles helpful in learning one another's professional roles and skills. They reported the prebrief helped them realize similarities in their practices even though differing terminology was used.

### 5.1 Limitations

The authors acknowledge this pilot study had some limitations including the use of a posttest only design. In future work we will add a pretest so we can examine the students' perceptions before and after the intervention. A second limitation was the small sample size of students from a single university. Future work will include larger numbers of interprofessional students with appropriate study power considerations.

## 6 CONCLUSION

Collaborative Sim‐IPEs can be time‐intensive to plan, schedule, and implement. They require expertise from differing course faculty to develop evidence‐based scenarios and require a commitment to finding time that is suitable for all learners to meet. However, our pilot Sim‐IPE successfully achieved the objectives to allow students from different healthcare professions to learn about one another's roles and responsibilities, collaborate as a team, communicate ideas, and listen to one another's perspectives. Introducing interprofessional collaboration to healthcare students is achievable and is key for continued successful work between professions. This is important for students from all healthcare professions to understand one another's scope of practice, roles, skills, and areas of expertise to improve outcomes for patients. A future simulation that will include healthcare students across multiple professions is currently being planned.

## AUTHOR CONTRIBUTIONS

*Conceptualization*: Jacqueline Vaughn, Robin Cunningham, Lindsey H. Schroeder, and Matthew J. Peterson. *Data curation*: Jacqueline Vaughn, Robin Cunningham, Lindsey H. Schroeder, Matthew J. Peterson, Stephanie Sims, Mia Rose Gambacorta, and Colette Waddill. *Quantitative analysis*: Matthew J. Peterson, Jacqueline Vaughn, and Lindsey H. Schroeder. *Qualitative analysis*: Jacqueline Vaughn, Colette Waddill, Stephanie Sims, and Mia Rose Gambacorta. Writing, review and editing: Jacqueline Vaughn, Robin Cunningham, Lindsey H. Schroeder, Matthew J. Peterson, Mia Rose Gambacorta, Stephanie Sims, and Colette Waddill.

## CONFLICT OF INTEREST

The authors declare no conflict of interest.

## Data Availability

The data that support the findings of this study are available on request from the corresponding author. The data are not publicly available due to privacy or ethical restrictions.
